# RP-HPLC method for simultaneous quantification of free and total thiol groups in native and heat aggregated whey proteins

**DOI:** 10.1016/j.mex.2020.101112

**Published:** 2020-10-17

**Authors:** Franziska Kurz, Claudia Hengst, Ulrich Kulozik

**Affiliations:** Chair of Food and Bioprocess Engineering, Technical University of Munich, Freising, Germany

**Keywords:** DTDP, Thiol reactivity, Disulfide concentration, Whey proteins, Heat denaturation

## Abstract

Disulfide formation of whey proteins during heat treatment via thiol oxidation is important with regard to techno-functional properties. Due to the formation of other oxidation products than disulfides, the decrease in free thiol concentration is not proportional to the disulfide formation. Thus, in order to evaluate thiol reactivity and disulfide concentration both parameters are required. Currently applied methods focus mainly on the loss of free thiols using the spectrophotometric Ellman's assay. Next to that, we improved an existing RP-HPLC assay using the thiol reagent 4,4′-Dithiodipyridine (DTDP) to quantify free thiols as well as total (free thiols and disulfide bonds) thiols of native and heat-treated whey proteins. Thereby, the sample preparation technique, the sample handling, and the analysis technique were optimized. Thus, the paper provides a simple RP-HPLC method for quantification of thiol oxidation reactions to determine heat-induced changes in the structure of whey proteins. In addition, the method should be applicable to other protein systems due to the method validation by proteins of different amounts of free and total thiols in their structure.

• Simple RP-HPLC method for quantification of free and total thiols using 4,4′-Dithiodipyridine (DTDP).
• High recovery rates for free and total thiols.
• High stability within 24 h.

## Graphical abstract

**Figure:**
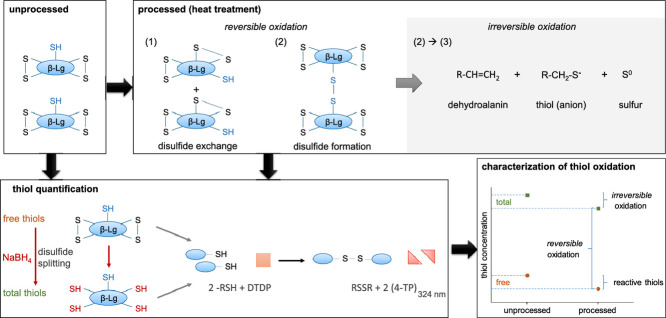

Specifications table

**Table:** 

Subject Area:	Chemistry
More specific subject area:	Thiol quantification
Method name:	RP-HPLC method for simultaneous quantification of free and total thiol groups in native and heat aggregated whey proteins
Name and reference of original method:	[1] Hansen, R. E., Østergaard, H., Nørgaard, P., & Winther, J. R. (2007). Quantification of protein thiols and dithiols in the picomolar range using sodium borohydride and 4,4′-dithiodipyridine. Anal. Biochem., 363(1), 77–82
Resource availability:	Reagents (Merck KGaA, Germany) • Hydrochloric acid (HCl) (37%) • Ethylenediaminetetraacetic acid (EDTA) • Sodium hydroxide (NaOH) • Sodium borohydride (NaBH_4_) • Guanidine hydrochloride (GdnHCl >99%) • Sodium phosphate dibasic (Na_2_HPO_4_) • Potassium phosphate monobasic (KH_2_PO_4_) • Tris(hydroxymethyl)aminomethane • 4,4′-dithiodipyridine (98%) (DTDP) • 1-octanol • L-cysteine hydrochloride (anhydrous, ≥98%) Materials • Syringe filter RC-45/25 Chromafil Xtra, pore size 0.45 µm (Macherey-Nagel GmbH & Co. KG, Germany) RP-HPLC • 1100 series (Agilent Technologies Deutschland GmbH, Germany) • Zorbax 300SB-C18-3.5 µm 4.6 × 150 mm (column) • Zorbax 300SB-C18-5 µm 4.6 × 12.5 mm (precolumn) • Agilent ChemStation software (Rev. B.04.03[16])

## Background information

Cysteine residues in proteins occur as the free thiol group or the oxidized disulfide cross-links and are known to be decisive for techno-functionality, sensory, and nutritional quality [2,3]. An important group of thiol-containing proteins are whey proteins, which are used in food structure design, e.g., in terms of creating gels stabilized by inter- and intramolecular disulfide cross-links [4], [5], [6]. The main whey proteins are β-Lactoglobulin (β-Lg) and α-Lactalbumin (α-La). α-La is absent of free thiol groups and contains four disulfide bonds [5,7], whereas β-Lg holds one free thiol group and two disulfide bonds in its structure [5,8]. The free thiol group of β-Lg, which is not accessible in the native protein structure, needs to be exposed to induce thiol oxidation and thus, the formation of disulfide bonds. This exposure via an unfolding of the tertiary protein structure can be achieved by exceeding a critical temperature (dependent on the medium about 60°C) [9]. Then, the free thiol group either interacts with an existing disulfide bond by a disulfide exchange reaction or with another free thiol forming a disulfide bond [10], [11], [12]. The latter results in a reduction of the amount of free thiol groups. The decrease in concentration of free thiols during processing, which is known as thiol reactivity, is thereby often used as an indicator to determine the formation of disulfide bonds. To monitor the decrease in free thiols, the spectrophotometric “Ellman's” assay is mainly used [14], [15], [13]. In brief, the sample is incubated with the thiol detecting reagent 5,5′-di-thiobis(2-nitrobenzoic acid) (DTNB), the so-called Ellman's reagent. Free thiol groups but not thiols interconnected in disulfide bonds react with DTNB, whereupon one equivalent of p-nitrothiophenol (NTP) per mol of free thiol groups is formed. As the NTP is detectable spectrophotometrically at 412 nm (molar extinction coefficient ε = 14,150 M^–1^cm^–1^ at pH 7.0) at a pH between 6.0 and 9.5, the concentration of free thiol groups can be quantified via the absorbance of the sample [1,^16^,17].

It is important to note that this approach neglects that free thiols do not necessarily form new disulfide bonds upon oxidation but multiple other oxidation products can result from the oxidation of the free thiol group, e.g., dehydroalanine residues and lanthionine [18], [19], [20], [21]. This means, a decrease in the concentration of free thiols is not proportional to the increase in disulfide bonds. Thus, the final concentration of disulfide bonds cannot be calculated from the reduction in free thiol groups (thiol reactivity) during processing.

**Fig. 1:**
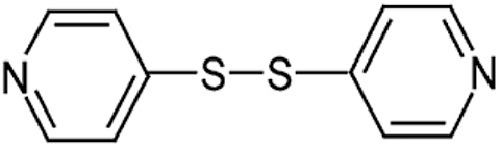
Structural formula of 4,4′-dithiodipyridine (DTDP) (adapted from [1]).

To identify the existence of disulfide bonds of native and heat-treated whey proteins directly, the reducing and non-reducing SDS PAGE (sodium dodecyl sulfate polyacrylamide gel electrophoresis) is performed most often. In this method, dithiothreitol (DTT) is used to reduce disulfide bonds before analysis. After electrophoresis, the proteins are stained and thus detectable by the formed bands [11]. Thereby, it is important to note that this is a semi-quantitative method. As an alternative, spectrophotometric assays, i.e. the Ellman assay, can also be used for quantification of reduced thiols. However, the disadvantage of spectrophotometry in general is the interference by components absorbing at the same wavelength contributing to the background absorbance at a specific wavelength. In this regard, a RP-HPLC (reversed-phase high performance liquid chromatography) method should be more suitable for this problem due to the possibility of a peak separation. Chen et al. [22] reported an HPLC assay based on the reaction of thiols with DTNB. Comparably to the Ellman's reagent, Hansen et al. [1] also reported a RP-HPLC assay for the quantification of free thiol groups and the total (free thiols and reduced disulfide bonds) amount of thiols of native proteins using the thiol detecting reagent 4,4′-dithiodipyridine (DTDP) as can be seen in Fig. 1.

The reaction is thereby based on the stoichiometric conversion of DTDP to 4-thiopyridine (4-TP), which absorbs at 324 nm [23]. Compared to the Ellman's assay using DTNB, the DTDP assay has an increased sensitivity due to the higher extinctions coefficient of the reaction product 4-TP (ε: 21,400M^−1^cm^−1^ at pH 7) compared to NTP [24]. In addition, the extinction coefficient is stable in the pH range from 3 to 7 and thus enables the detection of 4-TP by RP-HPLC operating at acidic pH values [1].

As a conclusion from the above, the purpose of this study was to establish a simple and accurate method to quantify the concentration of disulfide bonds as well as the concentration of the free thiol groups in native and heat-treated whey proteins to finally determine both, thiol reactivity and the amount of disulfide cross-linking.

Therefore, the method of Hansen et al. [1] was used as a basis. For the detection of thiol groups, Hansen et al. [1] incubated the sample in a buffer mixture consisting of the thiol detection reagent DTDP (0.36 mM), EDTA (0.2 mM) as a metal chelating agent, and the denaturation agent urea (6 M) to make the free thiol groups accessible to the reaction with DTDP. In addition, citrate (0.1 M) is used to adjust the pH to 4.5. After an incubation time of 30 min, DTDP is quantitatively converted to 4-TP, which is analyzed via RP-HPLC. A C-18 reversed-phase column was therefore operated isocratically with a 50 mM potassium acetate eluent at pH 4.0. Before determining the 4-TP concentration, a correction by the background signal (reagent blank in absence of protein) is required. Similar to the Ellman's reagent, DTDP cannot react with disulfide bonds. Thus, a cleavage of disulfide bonds by sodium borohydride (NaBH_4_) is required to analyze the amount of total thiols (free thiols and thiols bond in disulfide bonds). However, Hansen et al. [1] only carried out the quantification of free thiol groups and the total amount of thiols of native proteins (lysozyme, bovine serum albumin (BSA), RNase A, carboxypeptidase ϒ, and papain) and did not investigate heat-induced thiol oxidation of whey proteins.

In this regard, several adaptions to their method are needed to quantify thiol reactivity and disulfide cross-linking between proteins upon processing accurately and efficiently. Therefore, in brief, we improved the method by optimization of the composition of the reaction buffer and the used eluent to enable a fast, separated, complete, and time-independent (24 h) quantification of the reaction product 4-TP as shown in the method validation in detail. In addition, the demand of DTDP was adapted to the thiol content of the sample to reduce chemical consumption. Based on these changes, we provide a simple RP-HPLC method for quantification of free and total thiols of native and heat aggregated whey proteins using 4,4′-dithiodipyridine (DTDP). Thus, thiol reactivity can be determined by quantification of the loss of free thiols during heat treatment. In addition, the amount of disulfide bonds can be calculated by subtraction of the concentration of free thiols from that of the total thiols before and after heat treatment. The amount of irreversible formed oxidation products during heat treatment can be calculated by subtracting the concentration of total thiols after heat treatment from that before heat treatment.

To verify the method, proteins of varying amounts of free and total thiols in their native structure were analyzed. In order to assess cross-linking in the samples, a heating step was applied to expose the free thiol group in β-Lg and thus, to enable thiol oxidation.

However, this method should be applicable to other thiol containing protein samples for thiol and disulfide quantification and will be a useful analytical method in the study of thiol reactivity, disulfide formation, and degradation during processing. A method for verifying the method's accuracy for other proteins will be provided in the section of method validation.

## Method protocol

### Preparation and storage of 40 mM 4,4′-dithiodipyridine stock solution

• *No. 0*: 40 mM 4,4′-dithiodipyridine (DTDP) (Dissolve 0.40 mmol of DTDP in 80 µL of 37% HCl and fill up to a total volume of 10 mL with deionized water.)
• Store the solution in the dark and cold (4°C).

### Buffer and sample preparation for free thiols

#### Preparation of buffer solutions

• *No. 1*: 20 mM EDTA stock solution (Dissolve 0.4 mmol of EDTA in 10 mL of deionized water and adjust to pH 7 by using 1 M NaOH. Fill up to a total volume of 20 mL by using deionized water.)
• *No. 2*: 100 mM potassium dihydrogen phosphate (Dissolve 10 mmol of KH_2_PO_4_ in deionized water to a total volume of 100 mL.)
• *No. 3*: 100 mM sodium phosphate dibasic (Dissolve 10 mmol of Na_2_HPO_4_ in deionized water to a total volume of 100 mL.)
• *No. 4*: Na_2_PO_4_/ KH_2_PO_4_ buffer (Mix buffer *No. 2* and *No. 3* in such ratio that the resulting pH is 5.)
• *No. 5*: 6 M guanidine HCl/ 100 mM phosphate/ 0.2 mM EDTA buffer (Dissolve 600 mmol of GdnHCl up to 70 mL by using the KH_2_PO_4_ solution (*No. 2*). Add 1 mL of EDTA stock solution (*No. 1*). Add Na_2_HPO_4_ solution (*No. 3*) in small quantities until the pH value of the mixture is 5.0 . Fill up to a total volume of 100 mL with buffer *No. 4*.)

#### Calculation of the minimum required amount of 4,4′-dithiodipyridine (DTDP)

The minimum amount of DTDP, which has to be added to the sample, can be calculated by Eq. (1) with the expected concentration of free thiols c_RSH expected_ referred to a sample volume of 500 µL and the concentration of DTDP in the stock solution c_DTDP stock solution_ (*No. 0*). The value 0.6 is referred to the optimum required ratio of DTDP [µmol] to the expected thiols [µmol] in the sample solution.

(1) $$V_{DTDP\;stock\;solution}\left[\mu l\right]=\;\frac{0.6\cdot c_{RSH\;expected}\;\left[\mu mol\;\mu L^{-1}\right]\;\cdot 500\;\left[\mu L\right]}{c_{DTDP\;stock\;solution\;}\left[\mu mol\;\mu L^{-1}\right]}$$

The theoretical concentration of free thiols per protein-sample solution c_RSH expected_ can be calculated as shown in Eq. (2). c_i_ refers to the concentration of the respective protein i in a mixture of m proteins, its molecular mass M, and its amount n of free thiols. The respective molecular mass is calculated based on the amino acid composition of the respective protein.

(2) $$c_{RSH\;expected}\left[mol\;L^{-1}\right]=\sum_{i=1}^{m}\frac{c_{i}\left[g\;L^{-1}\right]}{M_{i}\left[g\;mol^{-1}\right]}\cdot n_{i}$$

#### Sample preparation

• Transfer 1500 µL guanidine-phosphate buffer including EDTA (No. 5) into a reaction tube.
• Add 500 µL sample solution and mix.
• Add the calculated amount of DTDP stock solution (compare Eq. (1)) and shake. Note: Short-time light exposure (outside of an amber HPLC vial) won´t affect the results.
• Wait 10 min until the reaction is completed.
• Check for pH 5.
  Note: The absorption coefficient of 4-TP is constant within the pH value of 3-7. If pH value is not 5, an adjustment of the pH has to be done by buffer *No. 2* or *No. 3*. The dilution of the sample by adjusting the buffer has to be considered.
• Transfer an aliquot of the solution into an amber HPLC vial.
• Determination of total thiol concentration by RP-HPLC by monitoring 4-thiopyridine (4-TP) at a wavelength of 324 nm (refer to Section Calibration and analysis of thiols by RP-HPLC)

### Buffer and sample preparation for free thiols and disulfide bonds (total thiols)

Note: In contrast to Section Buffer and sample preparation for free thiols, the total amount of thiols (free thiols and thiols of disulfide bonds) is analyzed with this method. Therefore, an additional disulfide bonds cleavage step has to be carried out to cleave the disulfide bonds).

#### Buffer preparation

• *No. 1*: (Section Buffer and sample preparation for free thiols): 20 mM EDTA stock solution (Dissolve 0.40 mmol of EDTA in 10 mL of deionized water and adjust to pH 7 by using 1 M NaOH. Fill up to a total volume of 20 mL by using deionized water.)
• *No. 6*: 750 mM potassium dihydrogen phosphate (Dissolve 75.0 mmol of KH_2_PO_4_ in deionized water to a total volume of 100 mL.)
• *No. 7*: 750 mM sodium phosphate dibasic (Dissolve 75.2 mmol of Na_2_HPO_4_ in deionized water to a total volume of 100 mL.)
• *No. 8*: Na_2_PO_4_/KH_2_PO_4_ buffer (Mix buffer *No. 6* and *No. 7* in such ratio that the resulting pH is 5.)
• *No. 9*: 6 M guanidine HCl/ 750 mM phosphate/ 0.31 mM EDTA buffer (Dissolve 600 mmol of GdnHCl up to 70 mL by using the prepared KH_2_PO_4_ stock solution (*No. 6*). Add 1.55 mL of EDTA stock solution (*No. 1*). Add Na_2_HPO_4_ solution (*No. 7*) in small quantities until the pH value of the mixture is 5.0. Fill up to a total volume of 100 mL with buffer *No. 8*.)

#### Preparation of solutions for the reduction of the disulfide bonds

• *No. 10*: 6 M guanidine HCl/ 0.5 M tris(hydroxylmethyl)aminomethane solution (Dissolve 50 mmol of Tris(hydroxylmethyl)aminomethane and 600 mmol of GdnHCl in deionized water to a total volume of 100 mL.)
• *No. 11*: Prepare a solution of 30% (w/v) sodium borohydride (NaBH_4_) using 1 M NaOH (Dissolve 40 mmol of sodium borohydride in 5 mL of 1 M NaOH).

#### Calculation of 4,4′-dithiodipyridine (DTDP)

The minimum amount of DTDP, which has to be added to the sample, can be calculated by Eq. (3) with the concentration of all thiols c_total thiols expected_ referred to a sample volume of 1,000 µL and the concentration of DTDP in the stock solution c_DTDP stock solution_ (*No. 0*). The value 0.6 is referred to the optimum required ratio of DTDP [µmol] to the expected thiols [µmol] in the sample solution.

(3) $$V_{\mathrm{DTDP}\;\mathrm{stock}\;\mathrm{solu}\mathrm{tion}}\left[\mu L\right]=\;\frac{0.6\cdot c_{\mathrm{total}\;\mathrm{thio}\mathrm{ls}\;\mathrm{expe}\mathrm{cted}}\;\left[\mu \mathrm{mol}\;\mu L^{-1}\right]\;\cdot 1000\;\left[\mu L\right]}{c_{\mathrm{DTDP}\;\mathrm{stock}\;\mathrm{solu}\mathrm{tion}\;}\left[\mu \mathrm{mol}\;\mu L^{-1}\right]}$$

The theoretical concentration of total thiols per protein-sample solution c_thiol expected_ can be calculated as shown in Eq. (4). c_i_ refers to the concentration of the respective protein i in a mixture of m proteins, its molecular mass M, and its amount n of total thiols after reduction by sodium borohydride (NaBH_4_). The respective molecular mass is calculated based on the amino acid composition of the respective protein.

(4) $$c_{\mathrm{total}\;\mathrm{thio}\mathrm{ls}\;\mathrm{expe}\mathrm{cted}}\left[\mathrm{mol}\;L^{-1}\right]=\sum_{i=1}^{m}\frac{c_{i}\;\left[g\;L^{-1}\right]}{M_{i}\left[g\;\mathrm{mo}l^{-1}\right]}\cdot n_{i}$$

#### Sample preparation

• Add 1000 µL of sample solution to 770 µL tris-guanidine solution (*No. 10*) and mix.
• Add 230 µL of freshly prepared sodium borohydride (*No. 11*) and mix.
• Put 50 µL of 1-octanol on top.
  Note: The 1-octanol prevents foaming caused by hydrogen formation.
• Incubate the mixture at 65°C for 60 min.
• Quench the reaction by addition of 400 µL of 5 M HCl. To do so, penetrate the 1-octanol layer and mix with the pipette.
  Note: NaBH_4_ is completely removed by acidification [1,25].
• Wait for 10 min until the reaction is completed.
• Add calculated amount of DTDP (compare Eq. (3)) and mix.
• Wait for 10 min until the reaction is completed.
• Filter the solution with a RC extra 0.45 µm syringe filter.
• Transfer 500 µL of the filtrate into an amber HPLC vial and add 1000 µL of guanidine-phosphate buffer (*No. 9*). Close the vial and mix well.
• Determine the total thiol concentration by RP-HPLC.

### Calibration and analysis of thiols by RP-HPLC

• *No. 12*: Prepare a 10 mM L-cysteine hydrochloride monohydrate stock solution (Dissolve 1 mmol of L-cysteine hydrochloride monohydrate in deionized water up to 100 mL.) for the preparation of the calibration standards.
• Dilute the stock solution *No. 12* to 5.0, 2.5, 1.0, and 0.5 mM with deionized water.
• Transfer 1850 µL guanidine phosphate buffer (*No. 5*) into reaction tubes and add 100 µL of each cysteine solution and 50 µL DTDP stock solution (*No. 0*) to each tube.
• Wait for 10 min until the reaction is completed.
• Transfer 1000 µL of the standard mixtures into amber HPLC vials.

**Fig. 2:**
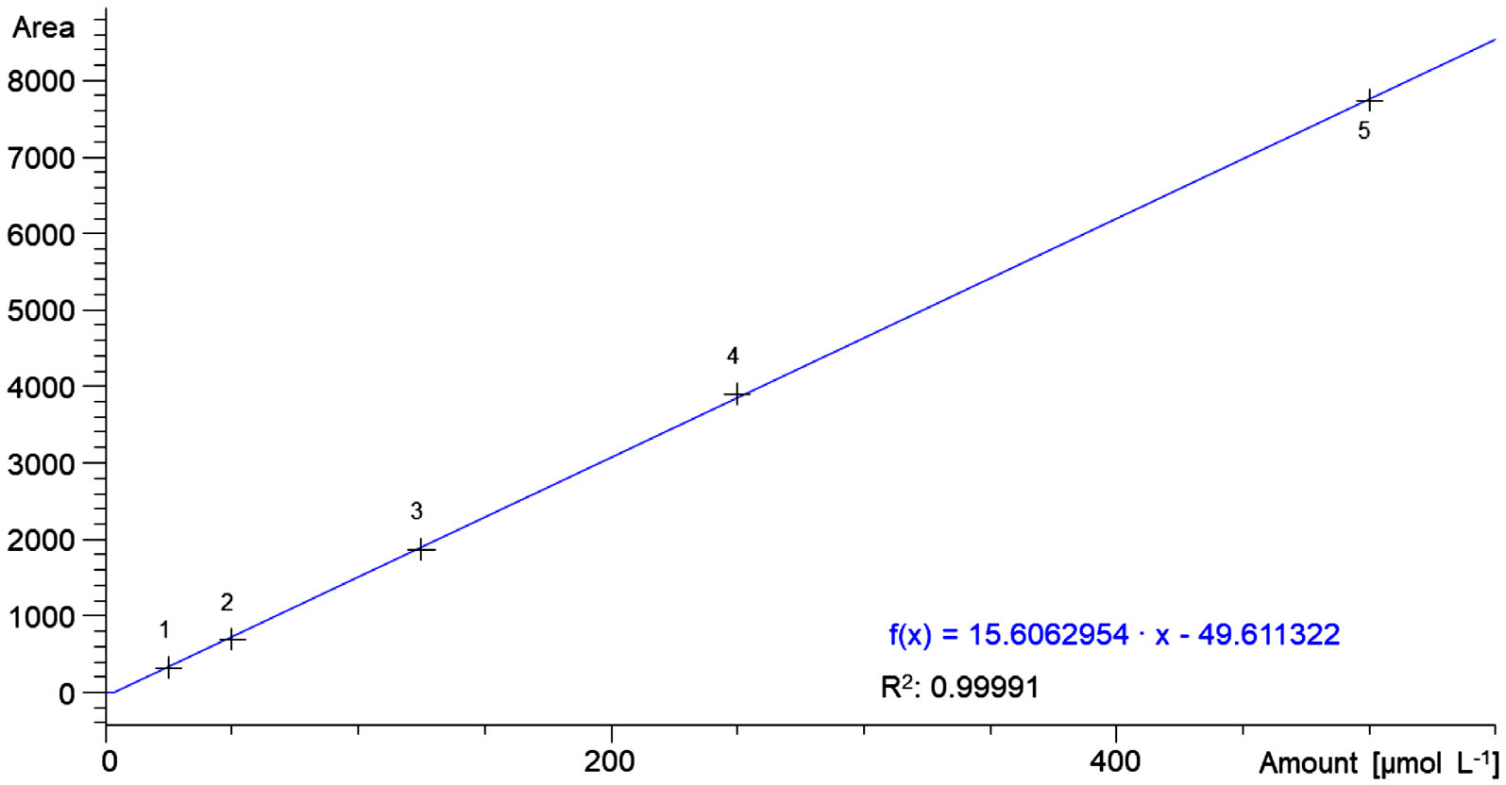
Peak area as a function of the amount of 4-TP in the cysteine standard solutions.

Analyze the standards by RP-HPLC using an Agilent 1100 Series (Agilent Technologies Deutschland GmbH, Germany) fitted with a Zorbax 300SB-C18-3.5 µm 4.6 × 150 mm column and a Zorbax 300SB-C18-5 µm 4.6 × 12.5 mm precolumn. Set the injection volume to 20 µL and the column oven temperature to 40°C. Gradient elution is performed according Table 1 at a flow rate of 1 mL min^−1^. A Diode-array-detector operating at 324 nm is used to monitor the absorption of the reaction product 4-thiopyridine (4-TP). The total duration of the analysis is 10 min. The peak areas are integrated by using the Agilent ChemStation software (Rev. B.04.03[16]). The standards of L-cysteine hydrochloride monohydrate are used to obtain a reference curve as shown in Fig. 2. It can be seen that the peak area is proportional to the 4-TP concentration (R^2^ = 0.99991).

**Table 1:** Gradient used for elution of 4-TP. Solvent A 100% gradient grade water containing 0.2% (v/v) trifluoroacetic acid (TFA); Solvent B 100% gradient grade acetonitrile (ACN) containing 0.2% (v/v) TFA.

Time	Solvent A	Solvent B	Flow
min	%	%	mL min^-1^
0.0	98.5	1.5	1.0
1.5	98.5	1.5	1.0
6.0	94.0	6.0	1.0
6.5	0.0	100.0	1.0
7.0	0.0	100.0	1.0
8.0	98.5	1.5	1.0
10.0	98.5	1.5	1.0

The analysis of the sample solutions is carried out accordingly. The amount of 4-TP in the sample is correlated to the peak area via the correlation function (Fig. 2).

### Calculation of parameters for characterization of thiol-disulfide reactions during processing

The quantified concentration of free (c_RSH_) and total (c_total_) thiols of unprocessed and processed proteins by using the described method can be used to calculate important parameters for characterization of thiol-disulfide reactions during processing.

The concentration of reactive thiols c_reactive thiols_ can be quantified by the decrease in concentration of free thiols during processing according to Eq. (5).

(5) $$c_{reactive\;thiols}\;\left[\mu mol_{SH}\;g_{protein}^{-1}\right]=\;\left(c_{RSH\;unprocessed}-\;c_{RSH\;processed}\right)$$

In addition, the amount of formed irreversible oxidation products during heat treatment can be calculated by subtracting the concentration of total thiols after processing from that before processing (Eq. (6)).

(6) $$c_{irreversible\;oxidation}\;\left[\mu mol_{SH}\;g_{protein}^{-1}\right]=\;\left(c_{total\;thiols\;unprocessed}-\;c_{total\;thiols\;processed}\right)$$

The knowledge on the concentration of free and total thiols before and after heat treatment is important for the calculation of the concentration of the disulfide bonds c_RSSR_ before and after processing. The concentration of disulfide bonds after heat treatment is influenced by the thiol reactivity but also by degradation of disulfide bonds. This is considered by using Eq. (7). The concentration of finally existing disulfide bonds (c_RSSR_) is thereby an important factor for the investigation of thiol-related protein aggregate properties such as the molecular flexibility in the context of stabilization of interfaces, e.g., adsorption and anchoring at interfaces [26].

(7) $$c_{RSSR}\;\left[\mu mol_{SH}\;g_{protein}^{-1}\right]=\;\left(c_{total\;thiols\;}-\;c_{RSH\;}\right)$$

## Method validation

The quantification of free thiols (RSH) by RP-HPLC is based on the detection of 4-TP (324 nm). Free thiols react with DTDP to 4-TP and a disulfide bond (RSSR) as follows from Eq. (8) [23].

(8) DTDP+2RSH→2(4−TP)+RSSR

To validate the method, the following protein powders were used:

• β-Lactoglobulin (β-Lg) A, β-Lactoglobulin B prepared from milk of cows homozygous for the A or B variant by skimming and production of whey protein isolates by membrane filtration (micro- and ultrafiltration in diafiltration mode) and subsequent isolation of β-Lg A and B, respectively, according to the method of Toro et al. [27] (purity >99%, determined by RP-HPLC)
• α-Lactalbumin (α-La) from bovine milk (purity ≥85% (polyacrylamide gel electrophoresis (PAGE)), Merck KGaA, Germany)
• Albumin from bovine serum (BSA) (purity ≥98% (agarose gel electrophoresis, Merck KGaA, Germany)
• Patatin purified from a commercial potato protein isolate powder (Solanic 200) with a high content of Patatin (AVEBE, The Netherlands) by preparative size exclusion chromatography using a Superdex 200 pg 26/600 (GE Healthcare, Germany) (purity >95% (PAGE))

### Buffer composition

For the analysis of free thiol groups, their accessibility is required to enable the reaction with DTDP, i.e., a complete unfolding of the proteins is decisive. According to the literature, guanidine HCl (GdnHCl) as a protein unfolding reagent is generally 1.5 to 2.5 times more effective per mole than urea [28], [29], [30]. This is probably of minor importance with native protein samples of small size as used by Hansen et al. [1]. However, aggregated gel-like structures are more resistant to dissolving and unfolding compared to native proteins. According to Dumpler et al. [31], a buffer containing 6 M GdnHCl is capable of completely unfolding native milk proteins as well as heat-denatured milk proteins in gel-like structures within a maximum time of 30 min. Since the method should be capable of completely unfolding highly denatured and aggregated structures within appropriate time, GdnHCl was chosen as denaturation reagent in contrast to urea used in the preceding method by Hansen et al. [1]. Therefore, it has to be tested whether the use of GdnHCl has a negative effect on the elution or the unfolding capacity and thus, the thiol recovery, respectively (Section Recovery rates and Thiol reactivity and disulfide formation/ degradation during heat treatment).

In order to provide an optimum peak separation (e.g. 4-TP, DTDP, protein) as well as to remove all materials from the column to improve the operation time of the reversed-phase C-18 column, a gradient elution using acetonitrile/ water mixtures was applied as recommended for RP-HPLC columns instead of 50 mM potassium acetate eluent used by Hansen et al. [1].

**Fig. 3:**
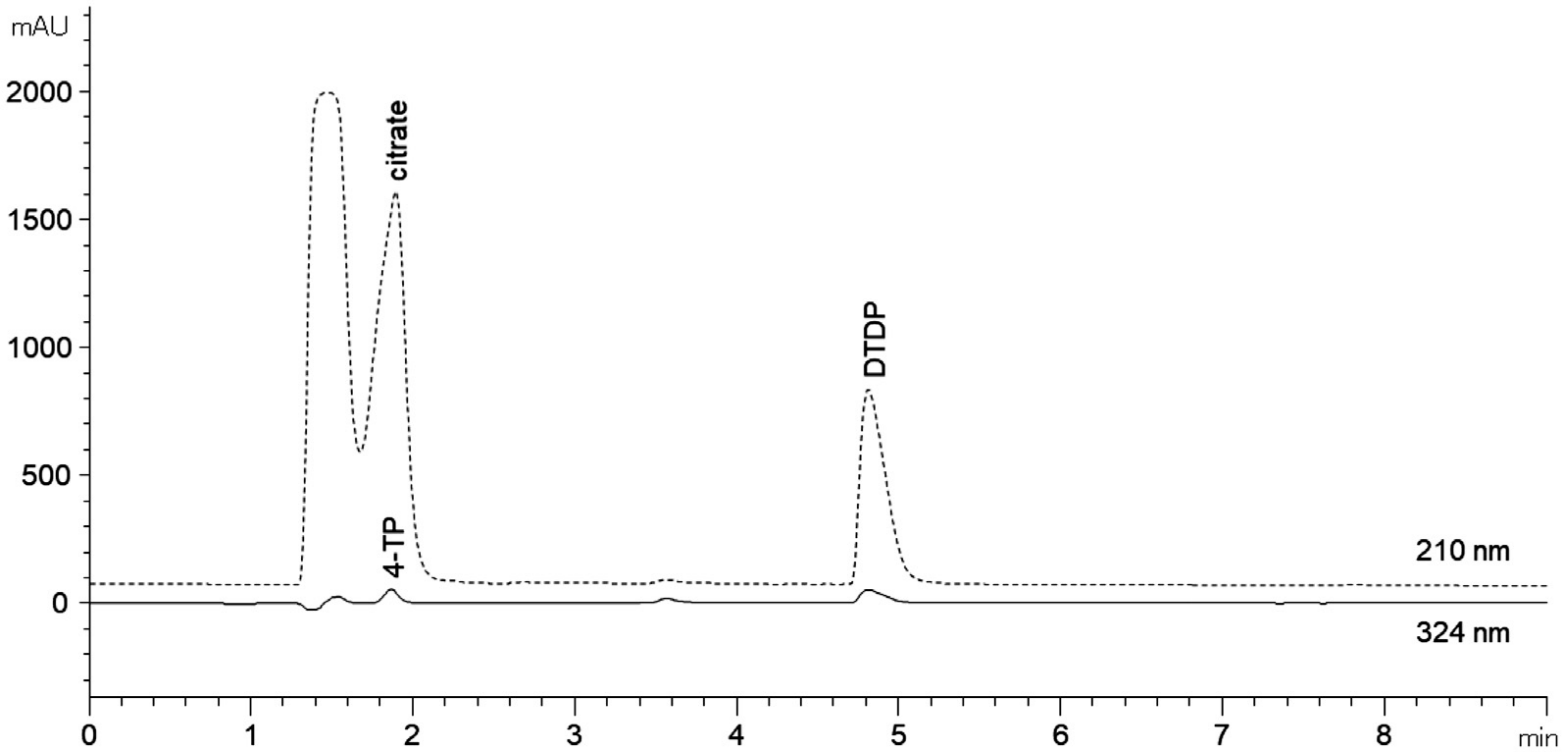
Chromatogram of a buffer system (GdnHCl, EDTA, and DTDP) containing citrate (pH 5) and 4-TP released by β-Lg at a wavelength of 210 (dashed line) and 324 nm (solid line).

To investigate the influence of GdnHCl and the acetonitrile/ water mixture on the elution, a buffer system containing GdnHCl (6 M) as a denaturation agent, EDTA, the thiol reagent DTDP, and β-Lg as a thiol containing sample to release 4-TP was prepared. The pH was adjusted to 5 using citrate. Thus, except for the GdnHCl, the buffer components were the same as used by Hansen et al. [1]. The chromatogram of the GdnHCl-buffer system at a wavelength of 210 and 324 nm is shown in Fig. 3.

**Fig. 4:**
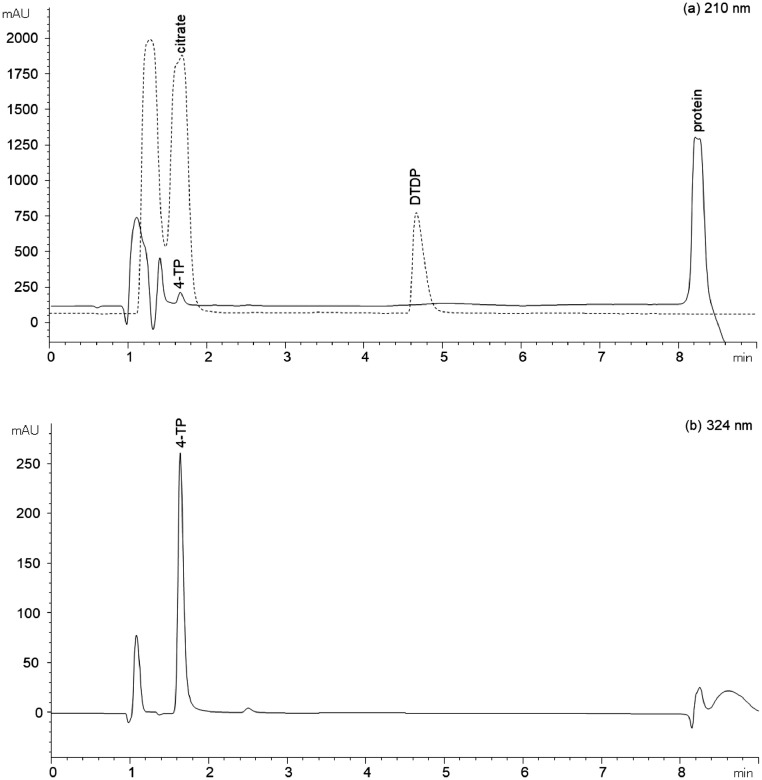
Chromatograms for the quantification of 4-TP at a wavelength of 210 (a) and 324 nm (b) using a reaction buffer consisting of GdnHCl, EDTA, DTDP, Na_2_HPO_4_, KH_2_PO_4_, and β-Lg to release 4-TP (solid line). As a guide to the eyes, the chromatogram of the GdnHCl, EDTA, DTDP, and citrate buffer (dashed line) is shown.

It can be seen that citrate and the reaction product 4-TP have the same retention time. Thus, the binding capacity of 4-TP on the column is interfered by citrate and this leads to peak deformation (chromatogram not shown) and consequently to limited quantification of 4-TP. Resulting from these findings, citrate was substituted by Na_2_HPO_4_ and KH_2_PO_4_ to adjust the buffer pH to 5. The sample preparation and analysis was performed as described in the Section Method protocol. As can be seen in Fig. 4, overlaying peaks can be observed at neither wavelength (solid line). The buffer blank (without protein) only exhibits the solvent peak (1 min) and thus, no interference by the blank is detectable (data not shown). In addition, the chromatogram of the buffer blank solution containing citrate is shown (dashed line). Thereby, the overlaying of the citrate peak over the 4-TP peak at 210 nm (and 324 nm) is quite clear to see (Fig. 4). Thus, using the modified buffer system containing GdnHCl, Na_2_HPO_4_, and KH_2_PO_4_ no interference on the elution can be detected.

Apart from peak overlaying, the elution of all retained materials is important with regard to the column operation time. As can be seen in Fig. 4a, all adsorbing buffer components (DTDP, 4-TP, and protein) are eluted from the column by using the acetonitrile/ water mixtures as an eluent. In consequence, an accumulation of buffer components on the column as well as a carryover of ingredients to the next run can be avoided. Thus, the used buffer-eluent combination ensures a high peak quality and a long column operation time.

The applicability of GdnHCl as an unfolding agent with regard to its effect on thiol accessibility of native and heat-aggregated proteins is shown in the Sections Recovery rates (native) and Thiol reactivity and disulfide formation/ degradation during heat treatment (aggregated proteins).

### DTDP concentration

Next to complete unfolding, a sufficient concentration of DTDP is required in order to quantify all free thiols. To avoid excess reagent consumption as well as to ensure quantitative detection of all existing thiols, the minimum required DTDP concentration has to be investigated. The concentration is thereby dependent on the amount of accessible thiols of the sample solution.

According to the reaction equation of DTDP (Eq. (8)), 1 mol DTDP reacts with 2 mol of free thiols forming 2 mol of 4-TP [23]. Thus, at least a ratio of 1 mol DTDP to 2 mol accessible thiols (RSH) is required to ensure complete quantification. The theoretical concentration of accessible thiols (free or total thiols) per protein-sample solution c_thiol expected_ can be calculated according to Eq. (2) or (4), respectively.

Consequently, the minimum required amount of DTDP is depending on the thiol content of the sample. In order to check whether a stoichiometric concentration of DTDP is capable to determine the total amount of free thiols, a β-Lg (A+B) solution (c_protein_: 9 g L^-1^) was used. Thus, the theoretical concentration of free and total thiols was calculated according to Eq. (2) and (4). As both β-Lg A and B contain one free thiol group and five thiols in total and have a molecular weight (M_w_) of 18.3 kg mol^-1^ [5], the expected concentration of free thiols and total thiols was 492 µmol L^-1^ (0.246 µmol per 500 µL) and 2459 µmol L^−1^ (2.459 µmol per 1000 µL), respectively.

**Fig. 5:**
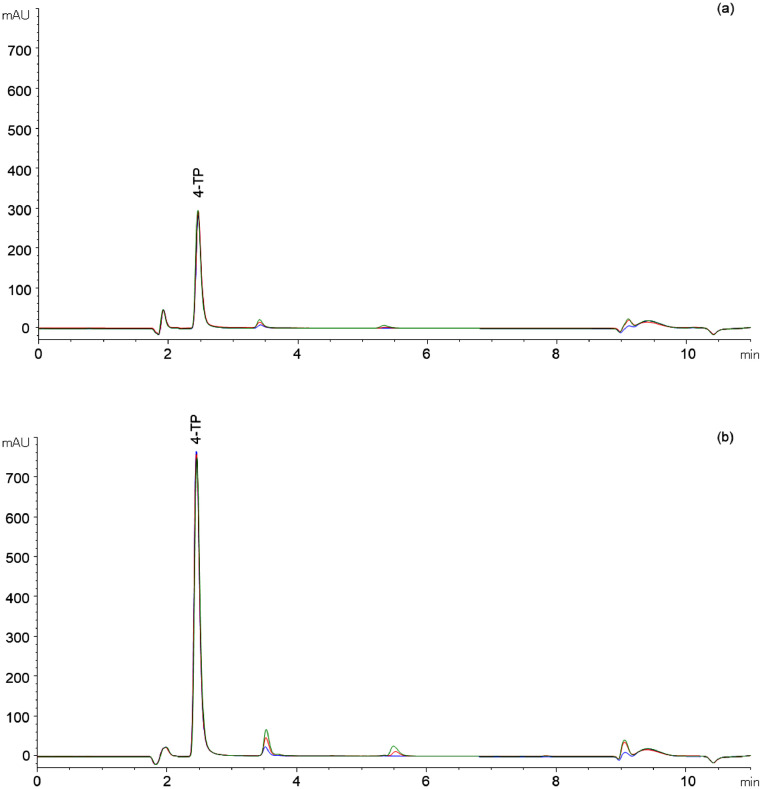
Chromatograms of β-Lg solutions (9 g L^-1^) for quantification of free (a) and total (b) thiols as a function of the DTDP [µmol]/thiols_expected_ [µmol] concentration ratio (0.5/1 (blue), 1.1/1 (red), 1.6/1 (green)).

According to the reaction Eq. (8), the minimum ratio of DTDP (µmol) to expected thiols (µmol_thiols expected_) is 0.5. Therefore, samples of different DTDP/thiols_expected_ ratios of 0.5 to 1.6 were prepared and analyzed as described in the Section Method protocol. The chromatograms are shown in Fig. 5. It can be seen that similar 4-TP peak areas (percentage variation_peak area_: <5% for free thiols and <0.3% for total thiols) occur for free as well as for total thiols, independently of the investigated DTDP/thiols_expected_ ratio. Thus, an excess concentration of DTDP did not result in a change in 4-TP concentration and thus in constant thiol concentration.

Apart from that, the concentration of 4-TP in the sample blanks (without protein) using also different amounts of DTDP (0.13 to 0.40 µmol and 1.24 to 4.00 µmol, respectively) was investigated. Due to the lack of proteins, the amount of 4-TP was expected to be zero. The concentration of 4-TP in the samples was below the detection limit of quantification for free thiols (area of 11.68) and total thiols (area of 26.69) independently of the added amount of DTDP. Therefore, a compensation of background absorbance is not necessary in that case.

Concluding from the above, a DTDP/thiols_expected_ ratio of 0.6 is recommended. In their method, Hansen et al. [1] used a constant concentration of 0.36 mM DTDP with more than 10 molar excess of 4-DTDP over thiol.

In addition, the unfolding capacity of GdnHCl as well as the stability of 4-TP in the solution after reaction is essential for sample handling. Therefore, we investigated whether a time-related change in the concentration of 4-TP was observable. To do so, the absorbance at 324 nm of all samples directly after preparation was compared to the absorbance of the same sample measured after 24 h (data not shown). Thereby, only a slight difference (< 1%) in the quantified 4-TP concentration within 24 h was detected. Consequently, GdnHCl is efficient to unfold native proteins within 10 min. In addition, at ambient temperature, 4-TP is stable for 24 h.

To conclude, within 24 h the detected concentration of 4-TP is independent of the DTDP/ thiols_expected_ ratio as long as this ratio exceeds 0.5. However, this has to be verified for each new sample system.

### Recovery rates

As shown above, the DTDP/thiols_expected_ ratio did not affect the 4-TP absorbance. However, in order to further establish the method, proteins of varying amounts of free thiols and disulfide bonds per molecule in their native structure were chosen for validation. Therefore, the recovery rates (Eq. (9)) for free and total thiols were calculated.

In detail, appropriate amounts of powder of the following proteins (Table 2) were dissolved in deionized water to a protein concentration of 9 g L^-1^ each (7.5 g L^-1^ Patatin) and stirred for 12 h at 4°C to ensure complete hydration.

**Table 2:** Recovery rates for free and total thiols of different whey proteins [5,32].

protein	M_w_	RSH_expected_	recovery_0h_	total_expected_	recovery_0h_
-	g mol^-1^	RSH molecule^-1^	%	RSH molecule^-1^	%
β-Lg A+B	18,320	1	95.12 ± 2.07	5	96.31 ± 5.88
BSA	66,267	1	*^1^	35	86.27 ± 0.82
α-La	14,161	0	*^2^	8	98.65 ± 0.18
Patatin	40,009	1	94.27 ± 1.89	1	94.43 ± 1.06

*^1^ not determined due to missing information regarding powder nativity; *^2^below detection limit

This was followed by the calculation of the expected free and total thiol concentration on the basis of Eq. (2) and (4). As shown in Table 2, the whey proteins β-Lactoglobulin (β-Lg), genetic variants A and B, and bovine serum albumin (BSA) include one free thiol group and two (β-Lg) or 17 (BSA) disulfide bonds in their native structure [5,8]. In contrast, α-Lactalbumin (α-La) is absent of a free thiol group and exhibits four disulfide bonds [5,7] whereas the potato protein Patatin includes one free thiol group and is absent of disulfide bonds [32].

(9) $$\mathrm{Reco}\mathrm{very}\;\mathrm{rate}\;\left[\%\right]=\;\frac{\mathrm{thio}\mathrm{ls}\;\mathrm{quan}\mathrm{tifi}\mathrm{ed}\;\left(\mathrm{RP}-\mathrm{HPLC}\right)}{\mathrm{thio}\mathrm{ls}\;\mathrm{expe}\mathrm{cted}}\cdot 100\%$$

The calculated recovery rates for each protein solution are shown in Table 2. It can be seen that high recovery rates of more than 94% for free thiols and for total thiols can be found with BSA as an exception. It has to be noted that the nativity of the β-Lg powder was 94% determined by RP-HPLC according to the method of Toro-Sierra et al. [27] and Dumpler et al. [31], whereas the nativity of the BSA and the α-La powder was unknown. As protein denaturation during powder production can cause irreversible thiol oxidation, lower values of free and total thiols might occur. This accounts for the deviation from the expected recovery rate of 100% and thus, the quantification accuracy is acceptable. In addition, the recovery rates of the free thiol group of Patatin for both sample preparation ways (free and total thiols) shows high and similar values. Thus, the method shows high accuracy regardless of the preparation way.

As already shown for β-Lg in the Section DTDP concentration, the 4-TP also shows a high stability within 24 h for BSA and α-La. The deviation in detected thiols within 24 h is thereby < 2%. Thus, the reaction can be performed from 10 min to 24 h after sample preparation. In addition, it can be concluded that GdnHCl is an effective unfolding reagent due to the high and time-independent recovery rate for free and total thiols of all investigated proteins.

### Reproducibility of the method

To validate the reproducibility of the method, a whey protein mixture (ideal whey) containing different proteins of variable amounts of free and total thiols was used. The reproducibility was assessed as follows: An ideal whey was produced by pH 4.6 precipitation of fresh raw milk and the subsequent filtration using a 0.45 µm filter to separate caseins and whey proteins. The whey protein composition and concentration in the filtrate was analyzed according to Dumpler et al. [31]. Based on this, the concentration of free and total thiols of the whey consisting of β-Lg (A+B), α-La, BSA, lactoferrin (17 disulfide bonds; M_w_ 82,400 g mol^−1^ [33,34]), and immunoglobuline G (16 disulfide bonds, M_w IgG2_ 150,000 g mol^−1^ [35]) was calculated according to Eq. (2) and (4). The recovery rates (Eq. (9)) were 82.11% ± 1.65% for the free thiols and 101.88% ± 1.67 for total thiols. The results for the 24 h analysis showed a deviation of less than 1.5% for concentrations of both free and total thiols.

Thus, the results clearly show that the method can also be used for mixtures of thiol containing proteins.

### Thiol reactivity and disulfide formation/ degradation during heat treatment

Thiol-disulfide exchange reactions of whey proteins mainly occur due to heat treatment. Thus, next to the quantification of free and total thiols of proteins in their native states, heat-treated whey proteins were investigated to further validate the method and to determine thiol reactivity and disulfide formation during heat processing.

To do so, a β-Lg solution (c_protein_ 8.8 ± 0.5 g L^-1^, pH 6.8) was heat-treated at 80°C for 90 min to expose the free thiol group and thus induce thiol-disulfide exchange reactions. In addition, the ionic strength was increased up to 40 mM by NaCl to increase the denaturation rate.

Table 3 shows the quantified thiol concentrations as well as the calculated concentrations of irreversible oxidation, reactive thiols, and disulfides before and after processing according to Eq. (5)–(7). Thereby, a decrease in the free thiol concentration of 23.10 ± 3.18 µmol_SH_ g_protein_^-1^ can be determined due to heat treatment. This corresponds an increase in thiol reactivity (c_reactive thiols_) of 23.1 ± 3.2 µmol_SH_ g_protein_^-1^. These results are in accordance with those found by Leeb et al. [14] for heat-treated β-Lg using the spectrophotometric Ellman's assay.

**Table 3:** Concentration of free and total thiols before and after heat treatment (80°C/ 90 min) of a β-Lg solution (pH 6.8, 40 mM NaCl) as well as the calculated amounts of irreversible oxidation products, reactive thiols, and disulfides.

Process	c_free RSH_	c_total_	c_irreversible oxidation_	c_reactive thiols_	c_RSSR_
-	µmol_SH_ g_protein_^−1^	µmol_SH_ g_protein_^−1^	µmol_SH_ g_protein_^−1^	µmol_SH_ g_protein_^−1^	µmol_SH_ g_protein_^−1^
unheated	53.40 ± 1.69	265.83 ± 14.07			212.43 ± 12.39
			28.35 ± 6.59	23.1 ± 3.2	
heated	30.30 ± 3.04	229.91 ± 1.41			199.61 ± 1.64

However, the concentration of total thiols decreased by 28.35 ± 6.59 µmol_SH_ g_protein_^-1^ after heat treatment. This difference in the total thiol concentration indicates the formation of irreversible oxidation products which cannot be reduced using NaBH_4_. An insufficient reaction time can be excluded due to the slight deviation (<5%) of thiol content within 24 h (data not shown). Regarding to the literature, degradation of disulfide bonds due to desulfuration (β-elimination) can occur during heat treatment, leading to the formation of dehydroalanine and persulfide. Further reactions between dehydroalanine and thiols result in the formation of irreversible oxidation products [18], which are not detectable by 4-TP due to the lacking reduction. According to Klostermeyer et al. [19] and Watanabe et al. [20], heat treatment of β-Lg solutions mainly results in the loss of sulfur due to the formation of dehydroalanine and further reaction products. Thereby, the extent of the loss of sulfur is increased at alkaline heating pH [19,36]. Thus, to further confirm the formation of irreversible oxidation products and to validate the method, solutions of β-Lg (A+B) (9 g L^-1^, 60 mM NaCl) were heated (80°C, 90 min) at pH 6.8 and 8.5 accompanied by quantification of total thiols before and after heat treatment. The concentration of irreversible oxidation products (c_irreversible oxidation_) was calculated by subtraction of the total thiol amount after heat treatment from that before heat treatment according to Eq. (6). Thereby, an increase in the concentration of irreversible oxidation from 33.3 ± 8.1 (pH 6.8) to 56.6 ± 0.5 µmol_SH_ g_protein_^−1^ (pH 8.5) products can be determined with increasing heating pH. Thus, the amount of detectable total thiols is decreased.

To sum up, the developed method has the advantage to quantify both, concentration of free thiols and disulfide bonds. This is important to describe free thiol oxidation due to the lacking proportionality of the increase in disulfide formation and the decrease in free thiol concentration during heat treatment. The described method can therefore be used for a separated consideration of thiol reactivity (Eq. (5)), formation of irreversible oxidation products (Eq. (6)), and the presence of disulfide bonds (Eq. (7)) during processing of proteins. Using the presented method, thiol oxidation reactions of whey proteins are determined more accurately as compared to the commonly used application of the Ellman's assay for determination of thiol reactivity and the semi-quantitative PAGE for disulfide formation. The knowledge on the concentration of existing disulfide bonds before and after processing is thereby crucial regarding the characterization of disulfide-induced changes in the molecular flexibility of proteins.

In addition, the described method should be applicable for other protein systems than whey proteins. However, the method has to be verified for each new sample system.
